# Hydrogen Controls the Heavy Atom Roaming in Transient Negative Ion

**DOI:** 10.1021/jacs.4c18446

**Published:** 2025-04-14

**Authors:** Smith Pataraprasitpon, Thomas F. M. Luxford, Roman Čurík, Jaroslav Kočišek, Dariusz G. Piekarski

**Affiliations:** † Institute of Physical Chemistry, Polish Academy of Sciences, 01-224 Warsaw, Poland; ‡ J. Heyrovský Institute of Physical Chemistry v.v.i., The Czech Academy of Sciences, Dolejškova 3, 18223 Prague, Czechia

## Abstract

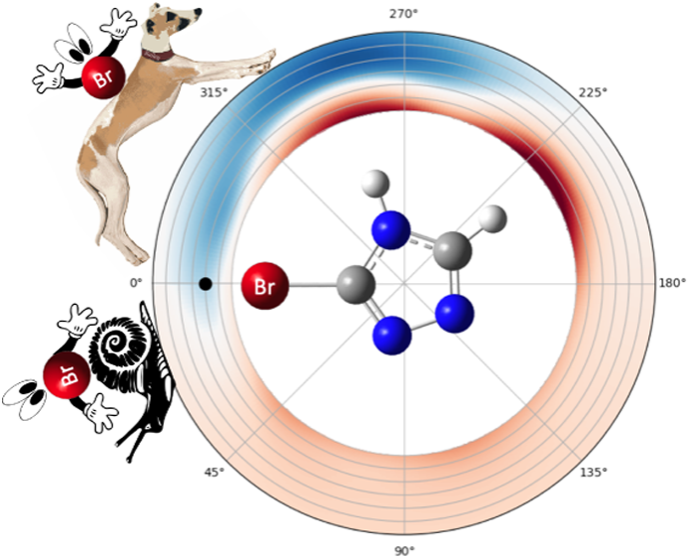

Bromine and hydrogen play unusual roles as mobile atom and dissociation dynamics moderator, respectively, during roaming in the 3-bromo-4H-1,2,4-triazole anion. The present study of the reactivity of 3-bromo-1H-1,2,4-triazole and 3-bromo-4H-1,2,4-triazole with low-energy electrons reveals the effect of the hydrogen position on the reaction dynamics. We report energy-dependent ion yields for both molecules showing significant differences. Quantum chemical calculations reveal that heavy Br atom migration is energetically more favored than H atom migration in the case of the H atom adjacent to Br. This is enabled by the energetically favorable formation of a noncovalent complex of Br^–^ around the triazole ring. Recently, such complexes have been reported for several other biologically relevant molecules. In the present work, we demonstrate that the position of hydrogen on the ring influences the character of the lowest resonant state and, consequently, the Br^–^ roaming and dissociation dynamics, particularly the neutral release of hydrogen bromide.

## Introduction

Imidazoles and 1,2,3 and 1,2,4-triazoles are common motifs in a variety of pharmaceuticals. The fact that triazoles physicochemically resemble the amide bond is widely used in the preparation of peptide analogs.^1^ Except for the amide bond, bioisosteres of esters and heterocycles are routinely prepared.^2^ Examples of applications include antibacterial, antifungal, and anticancer drugs and radiosensitizers. Further applications represent energetic materials, as the triazole ring stores a lot of energy.^3,4^

The use of azoles as antifungal agents in both medicine and agriculture raises significant concerns about their environmental effects. Upon disposal in water and soil, they become diluted to levels that are no longer effective against fungi. In this way, the fungi become more resistant to fungicides and highly resistant species appear.^5^ To stop this dangerous trend, techniques to remove azole-based antifungal agents from wastewater are under development.^6,7^ Methods based on decomposition are inefficient due to the high stability of the triazole ring. At the same time, the ring stores a large amount of energy. Proper trigger, therefore, can result in the release of this energy and decomposition of molecules, as it is used in azole-based energetic materials.

In energetic materials, the most common trigger is heat. Vibrational excitation results in hydrogen migration within the ring and isomerization that often decreases the decomposition thresholds of these compounds.^8,9^ Another trigger can be the low-energy electrons that decompose molecules in the dissociative electron attachment (DEA) process. For a hypothetical molecule AB, DEA can be written as AB + e^–^ → AB^#–^ → A + B^–^.

The attachment of a low-energy electron results in the formation of long-living transient negative ion AB^#–^, allowing for nuclear dynamics. The decay of the transient negative ion is then driven by the electron affinity of the decomposition products, representing a negative contribution to the reaction enthalpy. Electrons can therefore break bonds at subexcitation energies.^10^ Additionally, electron-induced isomerization and ring opening were demonstrated for highly stable ring molecules,^11,12^ including benzene.^13^ Processing of compounds with low-energy electrons, e.g., in low-temperature plasmas,^14^ therefore, may provide an efficient means of their removal from the environment. On the other hand, in the case of 1,2,4-triazoles, the ring appears to be stable upon electron attachment dominated by the decay of the ring-substituent functional groups.^15^

Regioisomerism plays an important role in the decomposition of 1,2,4-triazoles.^9^ Here, we explore the effect of dissociative electron attachment (DEA) reactions of two Br-substituted isomers, 3-bromo-1H-1,2,4-triazole (1HBrT) and 3-bromo-4H-1,2,4-triazole (4HBrT). The presence of the Br atom increases the electron attachment probability of the studied molecules and can facilitate electron transfer to the ring. Additionally, Br stabilizes two tautomers with respect to the hydrogen position, which is not possible for bare triazoles due to the fast hydrogen migration from position 4 to position 1.^8^

## Results and Discussion

**Figure 1:**
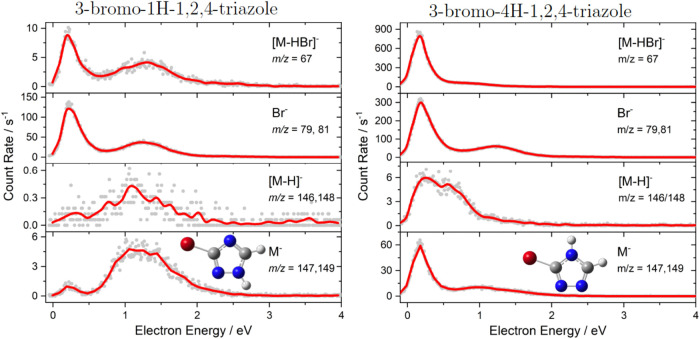
Energy-dependent anion yields for DEA to 3-bromo-1H-1,2,4-triazoles, 1HBrT and 3-bromo-4H-1,2,4-triazoles, 4HBrT. The solid line represents a 10 point average of the experimental data.

We applied electron attachment spectroscopy, irradiating the molecules with low-energy electrons (0–10 eV) of well-defined energy and analyzing the formed anions using mass spectrometry. Details can be found in the Methods section. The measured electron-energy-dependent yields for the most intense anions produced in DEA to 1HBrT and 4HBrT are shown in Figure 1. The curves for all detected fragment anions are present in Figures S1 and S2.

Density functional theory (DFT) predicts similar vertical attachment energies of 0.11 eV vs 0.08 eV for 1HBrT and 4HBrT, respectively. It is important to note that the DFT energies are influenced by the finite size of the base set used and the type of DFT functional employed. The small basis set is necessary to keep the resonant state bound. More advanced methods are suitable for the correct description of these states, such as the stabilization method, complex scaling potentials, and analytic continuation methods.^16−19^ In the present work, we applied the last of the mentioned methods to get more realistic positions of the resonances. The calculated values of the resonance positions are 0.244 and 0.197 eV using regularized analytic continuation (RAC)^18^ method and 0.251 and 0.201 eV using more precise barycentric analytic continuation (BAC) method^20^ for 1HBrT and 4HBrT, respectively. We can see that the analytical continuation energies are slightly above the DFT values, while they are in reasonable agreement with the experimentally determined values of 0.2 ± 0.1 and 0.15 ± 0.12 eV, respectively. The tiny differences in vertical attachment energies cannot describe the significant differences in the anion yields. The anion yields of 4HBrT are an order of magnitude higher than those of 1HBrT. This difference can be explained by two particular observations: (i) the resonance width calculated using the BAC method is 5 meV for 1HBrT, while it is only 0.1 meV for the 4HBrT, resulting in a much longer resonance lifetime than in the first case. (ii) The character of the resonant state shown in Figure S12, which in the case of 1HBrT is symmetric σ^*^ antibonding, indicates fast direct dissociation and asymmetric in the case of 4HBrT, indicating a propensity for anion rearrangement and more extended dissociation dynamics that will be further discussed in detail.

Both molecules form parent anions and the same set of fragments, even though the relative intensities of the fragments are different. Figure 1 shows that the most intense DEA channels arise from the breaking of C–Br or N–H bonds to form the parent minus hydrogen bromide [M – HBr]^−^, bromide Br^–^, and parent minus hydrogen [M – H]^−^ anions. However, the peak [M – HBr]^−^ is much more intense for 4HBrT, making this channel a dominant DEA in contrast to 1HBrT, which is dominated by the Br^–^ signal. The simple explanation of this surprising difference may be based on the proximity of the Br and H atoms in the case of 4HBrT, which allows for direct barrierless HBr formation and dissociation. However, this simple explanation does not correlate with significantly higher ion yields for the 4HBrT mentioned above. The differences can only be understood based on detailed reaction mechanisms and dynamics, showing easy Br^–^ migration.

The calculated reaction thresholds for the most intense reaction channels are listed in Table 1, and the corresponding structures are shown in Figure S2. The dissociation energies of 4HBrT are, in fact, lower than those of 1HBrT; however, the small differences of ∼0.25 eV are not enough to explain the significant differences observed in the fragmentation spectra. For example, for both molecules, the Br^–^ loss channel is exothermic, while the HBr exit channel is endothermic. Based purely on asymptotic energetics, Br^–^ should be the dominant fragment anion of both molecules. However, it dominates only 1HBrT fragmentation. Therefore, we assign these experimental observables to Br roaming in the 4HBrT anion. Roaming is a well-known phenomenon in photodissociation dynamics (see, e.g., refs (21−23)). However, roaming in dissociative electron attachment,^24,25^ or anions in general,^26^ is barely investigated in the literature.

**Table 1:** Dominant Relative Intensities of Anion Fragments Formed upon Electron Attachment to 1HBrT and 4HBrT and the Threshold Energies Calculated at the B3LYP/aug-cc-pVTZ Level of Theory^a^

	relative intensity, %			energy threshold, eV	
anion	1HBrT	4HBrT	neutral products	1HBrT	4HBrT
M^–^	9.2	10		0.04	0.04
[M – H]^−^	0.86	3.22	H	0.77	0.51
Br^–^	81.5	37.9	[M–Br]	–0.01	–0.04
[M – HBr]^−^	8	48.7	HBr	0.70	0.44

a See the Supporting Information for all of the data.

**Figure 2:**
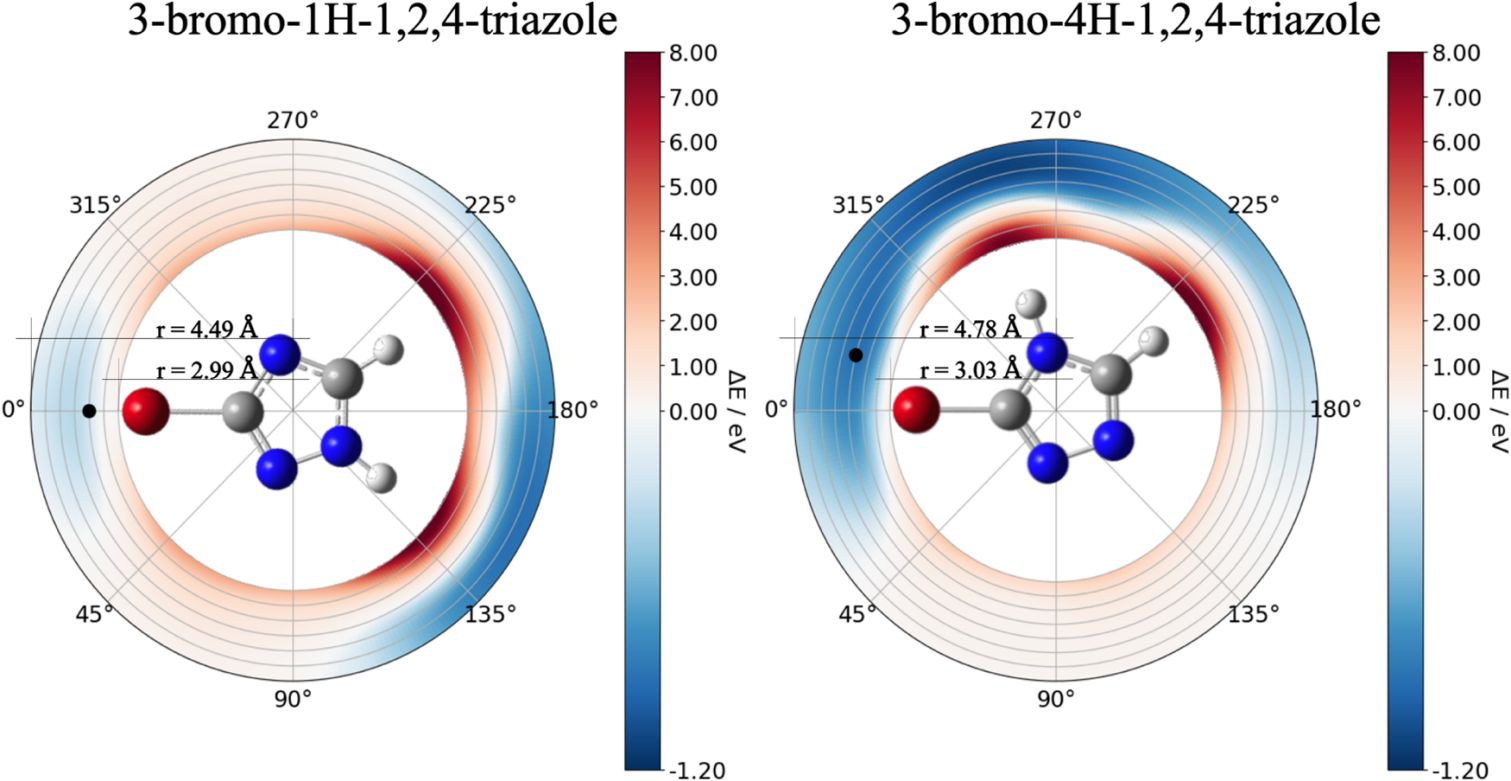
Energy landscape for Br roaming around the triazole ring. Relative electronic energies are in eV with respect to the adiabatic minima *m*/*z* = 147 at 0.04 eV for both molecules (see Figure 4 and the Supporting Information). The black dots correspond to the minima with *m*/*z* = 147 at −0.35 eV (see Figure S3) and −0.97 eV (see Figure 4) for 1HBrT and 4HBrT, respectively, with corresponding distances of ∼3.49 and ∼3.78 Å. The circles represent distances out of equilibrium distance (filled black dots) varying by −0.5, −0.25, 0.0, 0.25, 0.5, 0.75, and 1.0 Å; we included an extra distance of −0.75 Å for 4HBrT; see details for grid construction in the Supporting Information. Displacement of the bromine up to 1 Å out of the molecular plane does not affect the energy and charge landscapes as shown in Figures S9 and S10.

**Figure 3:**
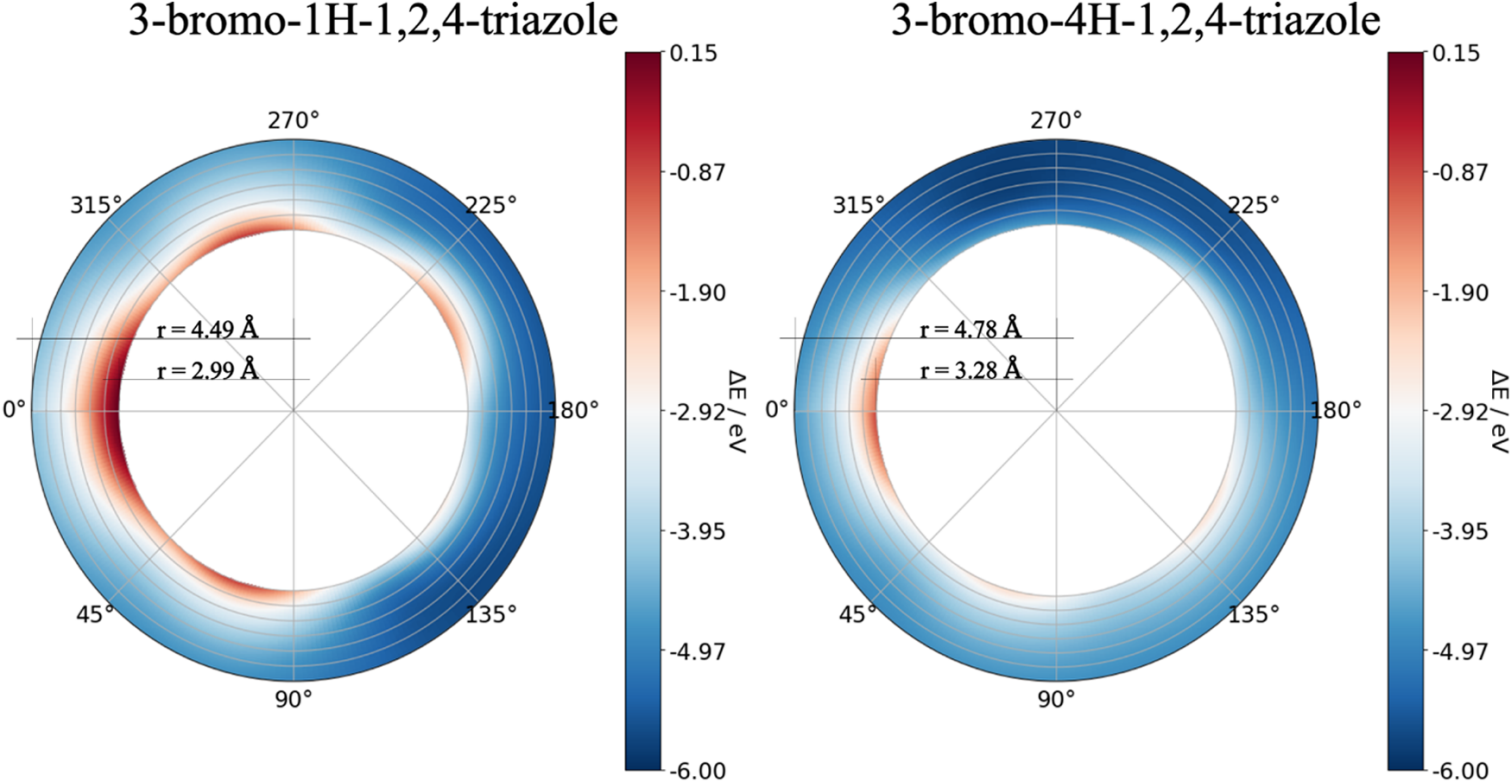
Qualitative analysis of electron autodetachment calculated in the vertical fashion as *E*^anion^ – *E*^neutral^ in eV at the B3LYP/aug-cc-pVTZ level of theory. Positive values mean that autodetachment is favorable for a given geometry of anion.

A 2D energy scan of the Br atom around the triazole rings is shown in Figure 2 (see details in the Supporting Information). In the case of 1HBrT, the Br^–^ can easily dissociate along the C–Br bond, while its migration clockwise or anticlockwise involves ∼0.7 eV (see 3D scan, Mulliken charge, and dissociation energy cutoff analysis in the Supporting Information). Straightforward exothermic Br^–^ mobility is observed for 4HBrT. Dissociation of Br^–^ is energetically suppressed at a longer distance (above 4.5 Å) at a 0° angle, while the heavy atom prefers to roam clockwise through the low-energy valley, getting trapped by the N–H-type hydrogen bond or even entirely around the triazole ring (up to ∼180°). This is in agreement with the 4HBrT resonant state’s singly occupied molecular orbital distorted by the proximity of the hydrogen atom (Figure S12). Even anticlockwise Br^–^ roaming is still more probable for 4HBrT than for 1HBrT (cutoff values of 0.23 and 0.34 eV, respectively; see details in the Supporting Information). Exploring the autodetachment probability over the roaming potential energy surface is interesting. We calculated vertical detachment energies in different anion geometries as shown in Figure 3. We can see that the electron is bonded stronger for 4HBrT. More importantly, only a very narrow region of autodetachment is possible for 4HBrT close to equilibrium, while 1HBrT can detach the electron in a wide range of geometries.

Formation of noncovalent complexes of Br^–^ has already been reported for several molecules and seems to play an important role in the dissociation dynamics of both isolated^25,27,28^ and solvated molecules.^29−31^ We can see that the complex formation slows down both fragmentation and electron autodetachment, which is in agreement with higher anion yields in the case of 4HBrT. In the present case, however, the complex formation does not influence only the anion lifetime and Br^–^ release but is actually also active in hydrogen and HBr release channels.

**Figure 4:**
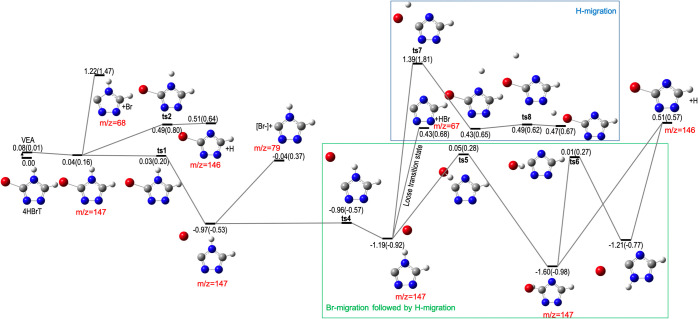
Potential energy surface for singly negatively charged 4H-bromo-1,2,4-triazoles, 4HBrT optimized at B3LYP/aug-cc-PVTZ. Relative energies in eV compared with the most stable neutral isomer of 4HBrT. DFT results are in agreement with coupled cluster; values in parentheses calculated at the CCSD/aug-cc-PVTZ//B3LYP/aug-cc-PVTZ level of theory.

To get better insight into the Br roaming mechanism and competing dissociation processes in the case of 4HBrT, we explored the potential energy surface (PES) shown in Figure 4. Upon vertical electron attachment (VEA), an anion at 0.04 eV is formed. We found a barrierless process via transition state **ts1** leading to a minimum at −0.97 eV, having a form of weakly bound Br^–^. Further, Br roaming involves much lower barriers than the Br^–^ dissociation itself (−0.96 eV vs −0.04 eV). Moreover, from the minimum at −1.19 eV, we found a barrierless process leading to neutral HBr emission via a loose transition state (see the Supporting Information). While a similar type of the noncovalently bonded complex could also be formed in the case of 1HBrT, it is a “dead end” for Br migration and the complex undergoes direct release of Br^–^. While 1HBrT primarily decays via autodetachment or dissociates via Br^–^ release, the 4HBrT forms a stable, noncovalent structure, allowing for much longer dynamics. Indeed, other processes, such as direct neutral Br followed by H_2_ molecule release, ring opening, and H-migration, are much higher in energy. Only the direct neutral H loss via **ts2** could compete with that of the neutral HBr loss channel. Interestingly, the noncovalent Br^–^ complex facilitates dragging the hydrogen atom by Br^–^ (see **ts5** and **ts6**), which should contribute to the experimentally observed yield of [M – HBr]^−^. Since the electron affinity of the HBr is 0.37 eV (calculated at the CCSD/aug-cc-PVTZ//B3LYP/aug-cc-PVTZ level of theory) and that of the remaining M – HBr is 3.26 eV, the charge can easily migrate to form neutral HBr and the observed *m*/*z* = 67 anion. Indeed, the natural population analysis for **ts5** and **ts6** transition states shows ∼0.1 e^–^ on HBr.

Losing a neutral H atom leading to the energy channel at *m*/*z* = 146 amu with 0.51 eV is energetically straightforward. On the contrary, the direct H-migration is significantly higher in energy, explaining the critical role of the Br on the reaction dynamics of the hydrogen 4-position. For exploration of higher-energy landscapes, see the Supporting Information.

Table 2 shows the relative channel population for both triazole targets obtained by molecular dynamics (MD) simulations. MD can detect the significant differences in the dynamics of 1HBrT and 4HBrT; however, it was not able to fully reproduce the experimentally observed anion yields. We assign the difference to the not sufficiently long simulation time scale (2 ps) in contrast to approximately 100 μs detection times in the experiment. First, we can see that the 1HBrT dissociation yield is dominated by Br^–^, while nearly no H-release is observed, in agreement with the experimental observation. Although the yields for 4HBrT are not consistent with the experiment, we can see that the direct Br^–^ release is not as important as that in the 1HBrT case. Instead, H and Br neutral release channels are observed with high intensities. These channels may be associated with the HBr release on the longer time scales. It is worth noting here that while the [M – Br]^−^ dissociation probabilities are similar for both molecules, the [M – H]^−^ channel strongly differs, being the most intense decay channel of the 4HBrT anion. MD can, therefore, correctly identify the importance of the H atom position in the dissociation process, even though it cannot reproduce the roaming dynamics and HBr formation step, which is the only energetically possible channel, considering the experimentally observed reaction thresholds.

**Table 2:** Relative Population in % of the Fragmentation Channels Obtained in the MD Simulations for All *E*_exc_ (0.01, 0.39, and 1 eV) for 1HBrT and 4HBrT (See Details in the Supporting Information)

	relative intensity, %	
anion	1HBrT	4HBrT
M^–^	29	0
[M – H]^−^	2	71
Br^–^	41	7
[M – Br]^−^	27	23

## Conclusions

Similar and simple halogenated 1,2,4-triazoles are different only by one H atom position 1H- vs 4H-, but they undergo different fragmentations after the DEA. In both molecules, two anionic states were identified based on *ab initio* modeling. The first is a short-lived one with a slightly negative electron affinity, and the second is a highly stable one. With this respect, the behavior is similar to that reported recently for several brominated cyclic hydrocarbons.^27,29^ However, the 1HBrT and 4HBrT anions undergo very different dissociation dynamics, determined by the position of the hydrogen atom. (i) The resonant state of the 1HBrT is highly symmetric, with a resonance width of 5 meV, while distortion of the bromine sigma antibonding orbital by the neighboring hydrogen atom in the case of 4HBrT results in a much smaller resonance width of 0.1 eV and longer anion lifetime. (ii) in 4HBrT, the bromine roaming can easily facilitate the H-release via the neighboring N–H activation in an exothermic process contrary to 1HBrT, where the energy threshold for Br moving toward any of H atoms is ∼0.8 eV. This impacts the fast direct Br^–^ release dominating the experimentally observed DEA yield of 1HBrT. In 4HBrT, the roaming dynamics result in a longer anion lifetime with respect to autodetachment and effective formation and release of HBr neutral.

## Methods

### Experiment

Fragmentation upon electron attachment to 3-bromo-1H-1,2,4-triazole (1HBrT) and 3-bromo-4H-1,2,4-triazole (4HBrT) was studied using a trochoidal electron monochromator-quadrupole mass spectrometer (TEM-QMS) apparatus.^32,33^ Molecules in the solid state were loaded to the direct-insertion probe of the spectrometer in a glass tube and sublimed without further heating (40 °C). The sample vapor passed through a 1 cm long, 0.3 mm diameter capillary into the reaction zone of the spectrometer, where the neutral molecules collided with electrons from the trochoidal electron monochromator (TEM). In TEM, electrons are emitted from the yttria-coated iridium stripe cathode and their energy distribution is narrowed using crossed electric and magnetic fields of the trochoidal monochromator.^34^ Such an electron beam is then accelerated to the desired energy in the reaction zone. Negative ions produced in the electron-molecule collisions were extracted from the reaction zone of the apparatus by ion optics, allowing for further focusing of the ions to quadrupole mass spectrometer, separating and detecting the ions according to their mass-to-charge ratio *m*/*z* ratio. The setup operates in constant energy or constant mass mode. In the first mode, fragment yields as a function of mass-to-charge ratio (*m*/*z*) are obtained at a constant energy of electrons. In the second mode, ion yields are obtained as a function of electron energy for a particular *m*/*z* fragment.

The samples were purchased from Merck and used without further purification. The stated purities of the molecules were 95 and 98% for 1HBrT and 4HBrT, respectively.

### Quantum Chemical Calculations

Potential energy surfaces, PES, of the fragmentation products of the 1HBrT and 4HBrT were calculated at the B3LYP^35−37^/aug-cc-PVTZ^38,39^ level of theory in Gaussian16.^40^ Single-point energies were computed for the selected part of PES at the CCSD^41,42^ level with the aug-cc-PVTZ basis set. Relative energies in eV were compared for each of the neutral molecules independently (1HBrT is 6.09 kcal mol^–1^/0.26 eV more stable than 4HBrT). Minima and transition states (TSs) were verified by frequency calculations. Connections between these saddle points were checked by intrinsic reaction coordinate (IRC) calculations.^43^ Geometries were also optimized at the B3LYP/def2-SVP^44^ level of theory, and energies improved by single-point (SP) energy calculations with the aug-cc-pVTZ basis set noted as B3LYP/aug-cc-pVTZ//B3LYP/def2-SVP. Finally, the energy thresholds were also compared at the B3LYP/def2-SVP low level of theory. Thus, we ensure that the geometries at the def2-SVP basis set are correct and its SP-corrected energies are in agreement with the results obtained at the B3LYP/aug-cc-PVTZ level of theory (see the Supporting Information for details). In this way, we verify the reliability of the relative population channels obtained in our molecular dynamics simulations. Molecular dynamics simulations were performed including the resolution of identity approximation and dispersion correction with the Becke–Johnson damping, i.e., RI-J^45^-B3LYP^35−37^/def2-SVP-GD3BJ^46,47^ levels of theory using the TURBOMOLE program.^48^ To mimic the dissociative electron attachment (DEA) experiments of 1HBrT and 4HBrT targets, we introduce the given amount of excitation energy E_*exc*_ randomly distributed over all of the nuclear degrees of freedom of the molecule for each trajectory and an extra electron in a Franck–Condon-type fashion. Thus, we run 100 trajectories for *E*_exc_ = 0.01, 600 trajectories for 0.39, and 200 trajectories for 1.00 eV per target molecule for up to 2 ps of the simulation time. In total, a reasonable 1800 trajectory sampling was obtained. The charges of the MD simulations were calculated using natural population analysis.^49^

## Supporting Information Available

Experimental energy-dependent ion yields for all DEA in the 0–4 eV range; experimental energy-dependent ion yields for CN^–^ DEA in a 0–12 eV range; detailed theoretical potential energy surface exploration; details of theoretical molecular dynamics exploration; benchmark calculations; and analytic continuation calculation for the lowest lying resonances (PDF)
