# Supplementary material for: Hydrogen Controls the Heavy Atom Roaming in Transient Negative Ion
Source: J Am Chem Soc. 2025 Apr 14;147(16):13370–6. doi: 10.1021/jacs.4c18446 (PMC12023026; doi:10.1021/jacs.4c18446)
Supplement: Supplementary file 1 — ja4c18446_si_001.pdf [file ja4c18446_si_001.pdf]

Supplementary Information: Hydrogen Controls the Heavy Atom Roaming in Transient Negative Ion

Smith Pataraprasitpon,[†] Thomas F. M. Luxford,[‡] Roman Čurík,[‡] Jaroslav Kočišek,[*,‡] and Dariusz G. Piekarski[*,†]

†*Institute of Physical Chemistry, Polish Academy of Sciences, 01-224 Warsaw, Poland*

‡*J. Heyrovský Institute of Physical Chemistry v.v.i., The Czech Academy of Sciences, Dolejškova 3, 18223 Prague, Czechia*

E-mail: jaroslav.kocisek@jh-inst.cas.cz; dpiekarski@ichf.edu.pl

Contents

1 Experimental details **S1**

1.1 DEA yields 0 eV - 4 eV, all fragments S1

1.2 DEA yields 0 eV - 12 eV, CN^- S2

2 Theoretical details **S3**

2.1 Potential Energy Surface Exploration S3

2.2 *Ab initio* Molecular Dynamics Exploration S11

2.3 Benchmark S13

2.4 Analytic Continuation Calculation for the Lowest Lying Resonances S15

1 Experimental details

1.1 DEA yields 0 eV - 4 eV, all fragments

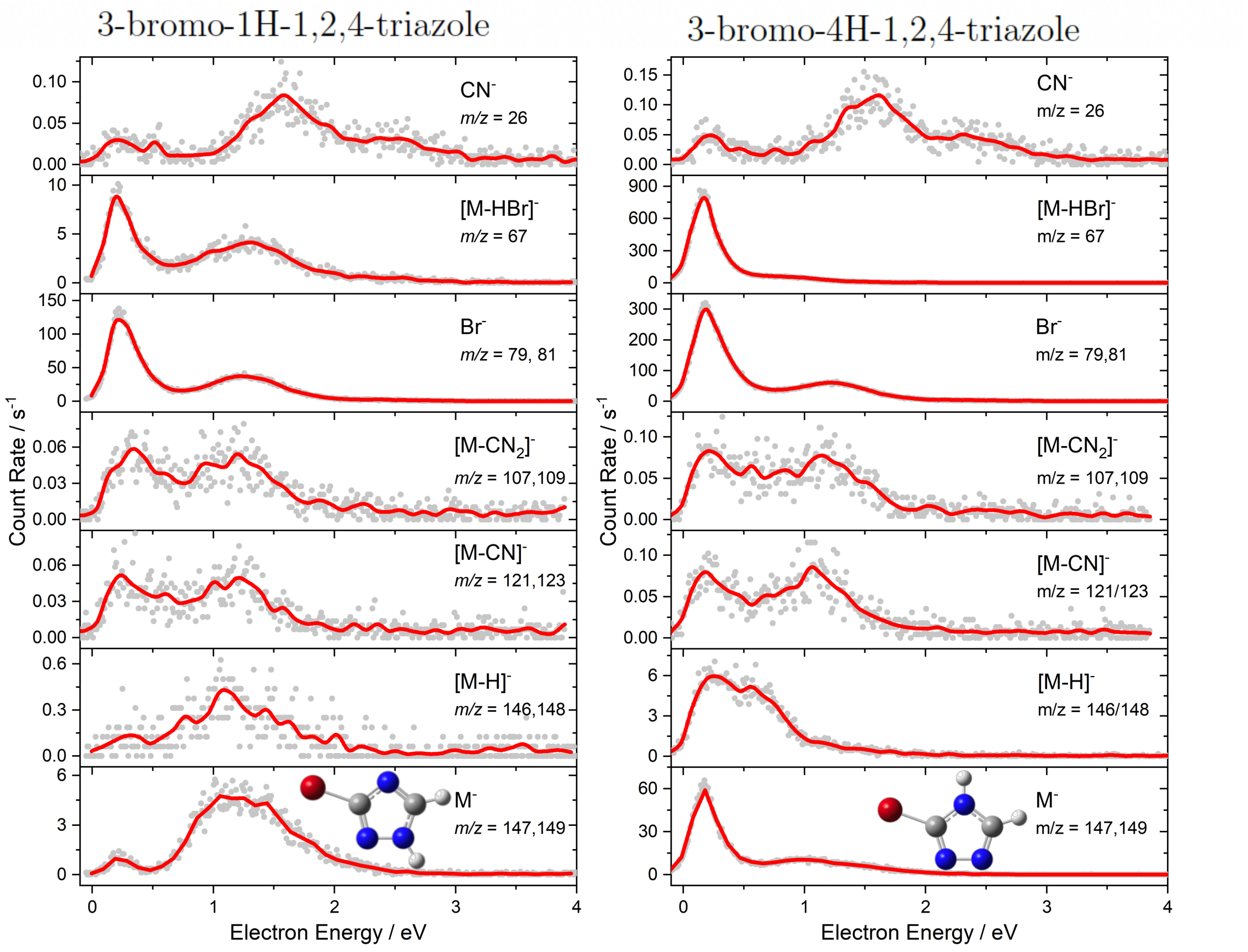

Figure S1: Energy dependent ion yields for particular anion reaction products of dissociative electron attachment to a) 1HBrT and b) 4HBrT in 0 eV - 4 eV electron-energy range.

1.2 DEA yields 0 eV - 12 eV, CN^-

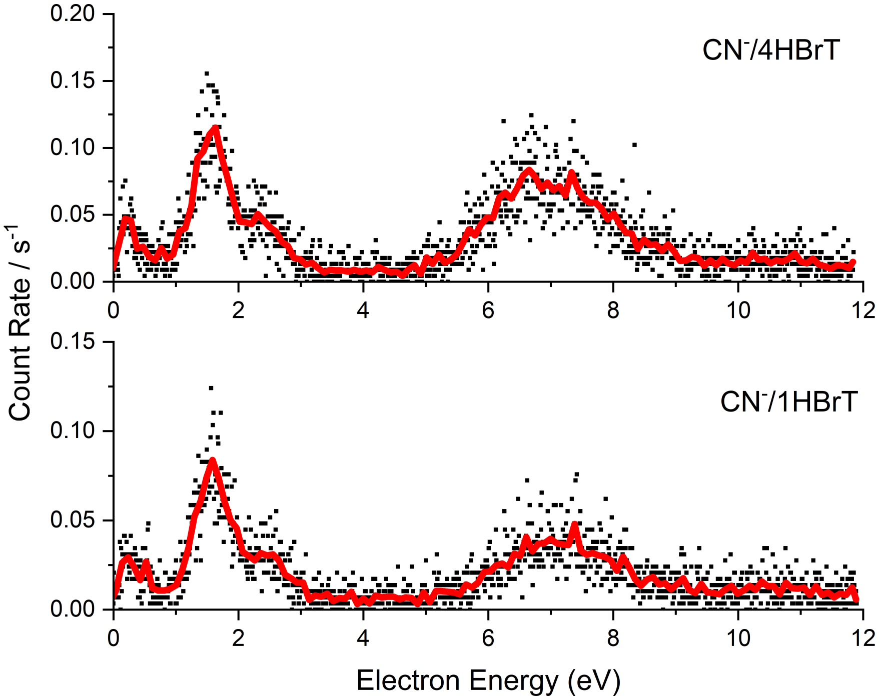

Figure S2: Energy dependent ion yields for CN^- formation upon dissociative electron attachment to a) 1HBrT and b) 4HBrT in 0 eV to 12 eV electron-energy range.

2 Theoretical details

2.1 Potential Energy Surface Exploration

Potential energy surfaces were calculated, accounting for electronic and zero-point energy corrections. The results are presented as relative energies with reference to the most stable neutral conformer of either 3-bromo-1H-1,2,4-triazole or 3-bromo-4H-1,2,4-triazole. All geometry and energies are obtained with B3LYP functional with aug-cc-pVTZ and def2-SVP basis sets. The low-level basis set performs poorly but captures the relative trend for energy threshold channel openings between both targets overall.

Fig. S3 shows the structures and energies of the most important peaks in the DEA experiments for a) 1HBrT and b) 4HBrT fragmentation. The most stable fragments do not correlate well with relative intensities of the peak observed in the experiments because it does not account for the kinetic barrier, which involves significant barriers or barrierless processes in the given channel production. For simple geometrical reasons, the Br atom is involved in the H-loss only for the 4HBrT, resulting in the fragmentation at 0.44 eV. H-loss from carbon position 5 is significantly higher in energy (2.34 eV). For 1HBrT molecule the Br can not be directly involved, the energy of the corresponding exit channel is 0.28 eV higher than for 4HBrT; H-loss from position 5 lies at 2.25 eV.

Fig. S4 shows the full PES that we considered for singly negatively charged fragmentation of 1HBrT. Interestingly, the presence of Br stabilizes the triazole ring after H_2 release via **ts1** transition state at 1.22 eV. On the contrary, while the neutral Br dissociates, we see that subsequent H_2 migration leads to ring opening and linear weakly bounded minimum at 1.70 eV. Finally, the endergonic loss of H_2 forms the minimum at 1.43 eV. However, H_2 loss after Br loss involves high barrier **ts5**=5.38 eV and should not be observed at current DEA experiments. Similarly, the Br or Br^- release after H_2 release should be thermodynamically not possible (see minima at 4.56 and 5.26, respectively).

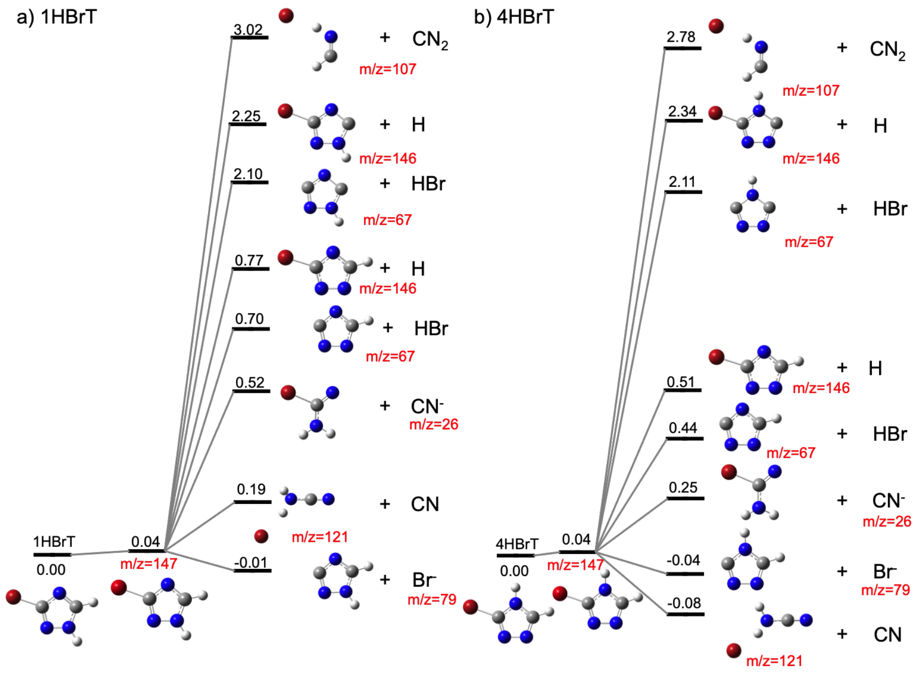

Figure S3: Potential energy surfaces of the most observed experimental peaks formed upon electron attachment to a) 1HBrT and b) 4HBrT. Relative energies to each neutral target in eV are calculated at the b3lyp/aug-cc-pVTZ level of theory.

Fig. S5 shows the full PES that we considered for singly negatively charged fragmentation of 4HBrT. First, it is seen that the H-migration after neutral Br loss demands 3.50 eV (see **ts9**) and should not be accessible in the experiments. Similarly, high exit channels are for m/z=67 and 79, while the first step is neutral H-atom loss. All possible neutral H-loss from different isomers of 4HBrT are much higher in energy than the channel at 0.51 eV. Ring opening of 4HBrT before fragmentation seems also possible, since it involves the barrier **ts3** below 1 eV, leading to a linear minimum at 0.50 eV. Interestingly, the loss of H_2 and Br also follows the opening of the ring that forms the linear minimum at 1.43 eV. There is no experimental evidence for the formation of this anion, however, it is worth saying that all the products created by ring decomposition are formed at very low intensities. This is in agreement with previous observation that the triazole ring is highly stable against LEE-induced decomposition.[1] Fig. S6 shows the relaxed scan along the N-Br bond starting at minimum at -1.19 eV in Fig. S5. Barrierless neutral HBr release is observed with ∼1.1 eV

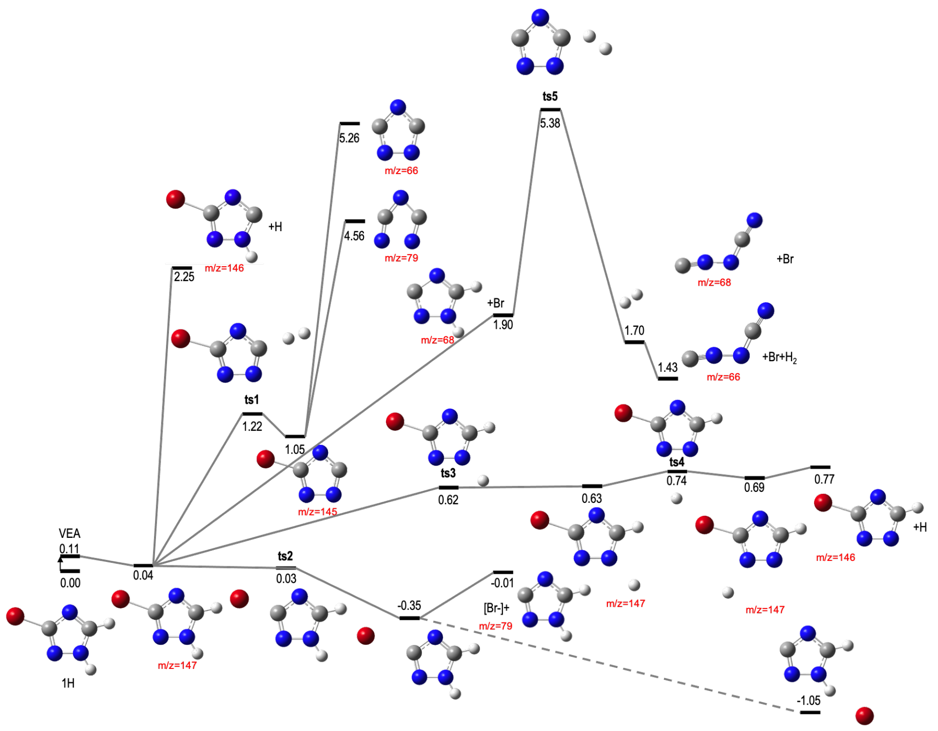

Figure S4: Potential energy surfaces for singly negatively charged 1H-bromo-1,2,4-triazoles, 1HBrT. Relative energies in eV compared with the most stable neutral isomer of 1HBrT.

loose transition state energy.

The grid for the 3D-scan was build starting with the center of the triazole ring defined as a center of the plane of triazole ring. Then we placed the Br in that plane at the distance that corresponds to the distance from the center of the ring to the Br for the corresponding minimum of 1HBrT and 4HBrT i.e. the black dots in Fig. S7 with m/z=147 at -0.35 eV (see Fig. S4) and -0.97 eV (see Fig. S5) for 1HBrT and 4HBrT, respectively with corresponding distances of ∼3.49 and ∼3.78 Å. We moved Br by -0.5, -0.25, 0.25, 0.5, 0.75 and 1 Å from the center of the plane, as well as along 2π radians by 45° covering 2D space; -0.75 Å distance was additionally covered for 4HBrT. Finally, we moved the Br along the vector perpendicular to the plane by 0.25, 0.5, 0.75 and 1 Å per each distance and angle, resulting in 3D-scan of 840 and 960 grid points per 1HBrT and 4HBrT, respectively. The single point energies were calculated at b3lyp/aug-cc-pvtz level of theory for each grid point. Multiquadratic radial basis functions $\phi(r) = \sqrt{(r/c)^2 + 1}$ were used to interpolate between grid points. The

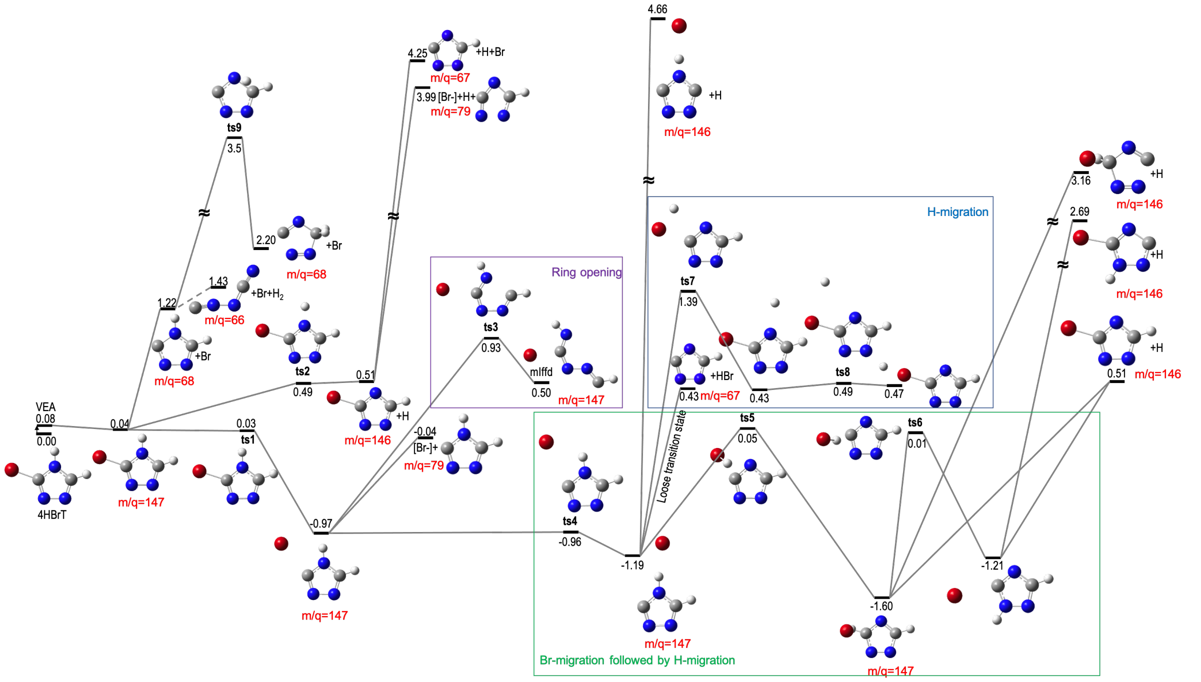

Figure S5: Potential energy surfaces for singly negatively charged 4H-bromo-1,2,4-triazoles, 4HBrT. Relative energies in eV compared with the most stable neutral isomer of 4HBrT.

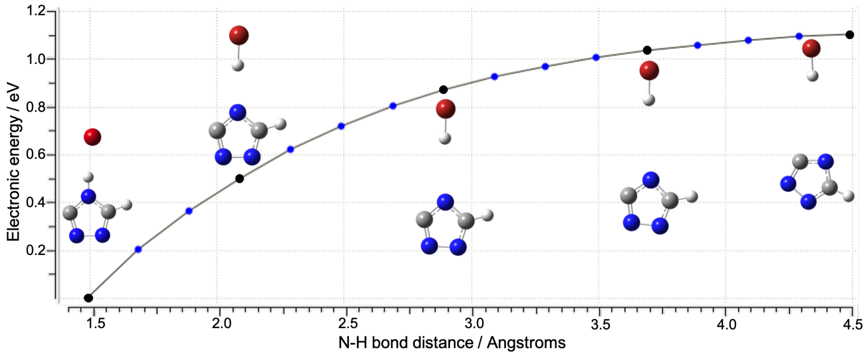

Figure S6: Relaxed energy scan for N-H bond distance showing the barrier-less HBr release i.e. loose transition state at 1.1 eV in respect to minimum at 0.0 eV (first black point).

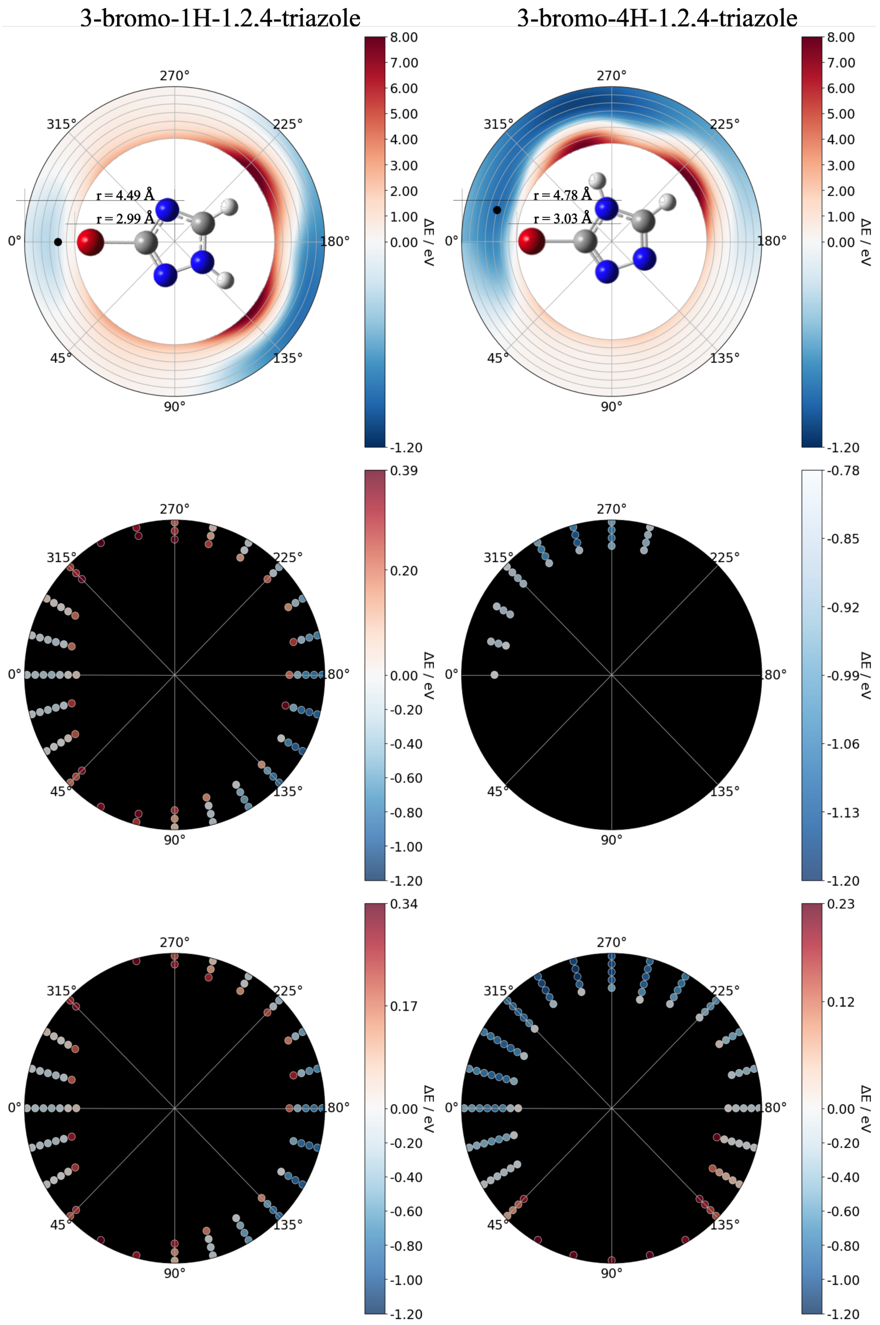

Figure S7: Energy landscape for Br roaming around the triazole ring. Relative electronic energies in eV in respect to the adiabatic minima m/z=147 at 0.04 eV for both molecules (see Fig. S4 and Fig. S5). The black dots correspond to the minima with m/z=147 at -0.35 eV (see Fig. S4) and -0.97 eV (see Fig. S5) for 1HBrT and 4HBrT, respectively with corresponding distances of ∼3.49 and ∼3.78 Å. For 1HBrT, Br roaming is always endothermic and has an energy cutoff of 0.39 eV to move clockwise and an energy cutoff of 0.34 eV to move anticlockwise. In contrast, for 4HBrT, the energy cutoffs are significantly lower. Br roaming is an exothermic process (-0.78 eV energy cut-off) to move clockwise and 0.23 eV in the opposite direction. These values highlight the differing dynamics of Br movement between the two triazole tautomers.

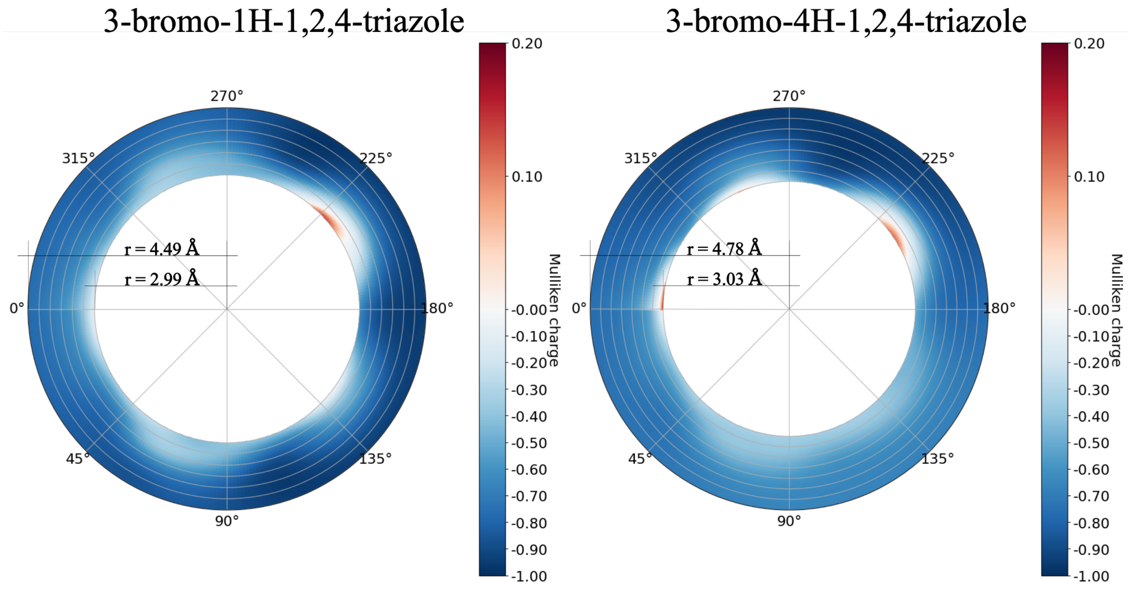

Figure S8: Charge landscape shows bromine (Br) roaming around the triazole rings. The negative charge generally tends to localize on the Br atom while out of C-Br equilibrium distance; Br is more negative as the distance from the center of the ring increases, especially in the N-H and C-H neighborhoods. These trends are very similar in case of both molecules.

resulted "donut" plots were prepared for energy and charge scans.

In Fig. S7 we see the energy landscape around the triazole ring for a given set of distance. Br roaming is endotermic process only for 4HBrT. For 1HBrT in order to move Br atom around the ring we need to deliver at least 0.34 eV (see energy cut-off on the energy scale).

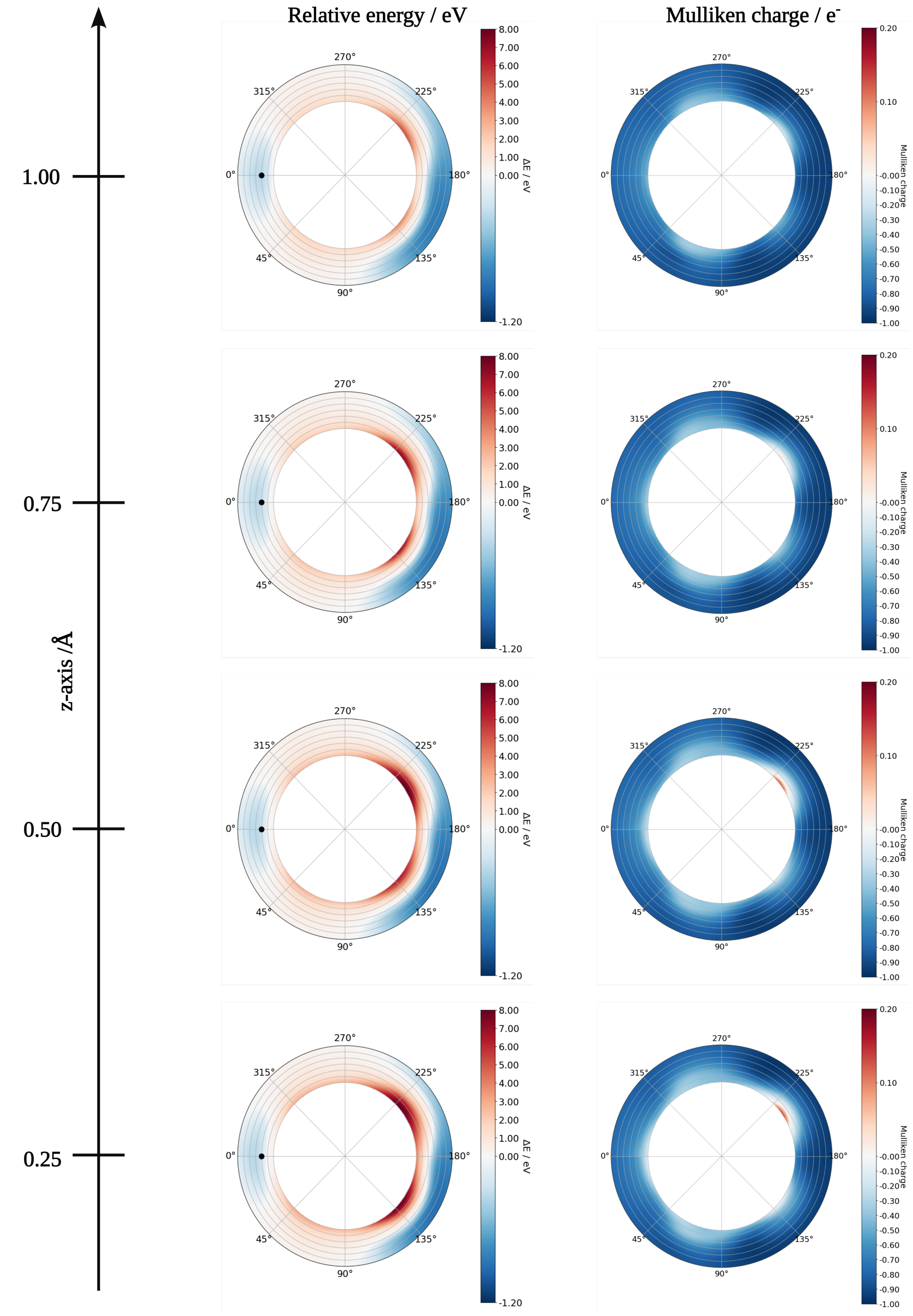

Figure S9: The plots depict the variations in relative energy and Mulliken charge for 1HBrT from bottom to top as the distance from the molecular plane increases incrementally from 0.25 to 1.00 Å by 0.25 Å step for 3-Bromo-1H-1,2,4-triazole, illustrating that the energy and charge distributions are not affected by the out-of-plane displacements up to 1 Å.

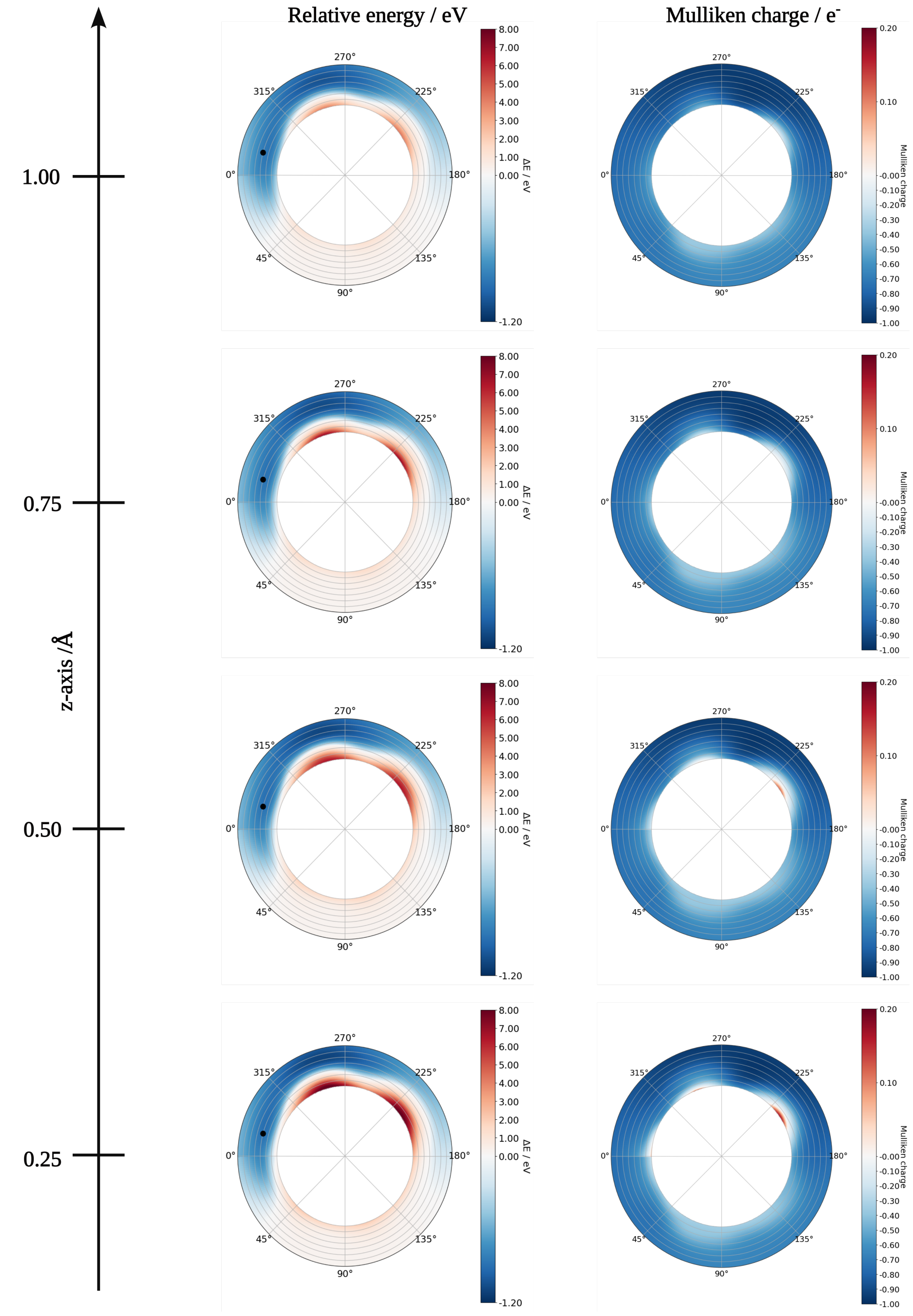

Figure S10: The plots depict the variations in relative energy and Mulliken charge for 4HBrT from bottom to top as the distance from the molecular plane increases incrementally from 0.25 to 1.00 Å by 0.25 Å step for 3-Bromo-1H-1,2,4-triazole, illustrating the the energy and charge distributions are not affected by the out-of-plane displacements up to 1 Å.

2.2 *Ab initio* Molecular Dynamics Exploration

To mimic the electron attachment of the bromo-1,2,4-triazole derivatives we introduce the given amount of excitation energy E_{exc} randomly distributed over all the nuclear degrees of freedom of the molecule for each trajectory and attached one electron in a Franck-Condon type transition. Finally, we run 100 trajectories for E_{exc} = 0.01 eV, 600 trajectories for E_{exc} = 0.4 eV and 200 trajectories for E_{exc} = 1 eV, performing in total a reasonable 900 trajectory sampling per each target molecule (1800 in total). All the molecular dynamics simulations are performed in TURBOMOLE[2] using the resolution of the identity approximation[3] and dispersion corrected with with Becke-Johnson damping[4,5 6–8]/def2-SVP[9] level of theory. The charges for the MD simulations were calculated using natural population analysis.

Figure S11 shows the results of all 1800 trajectories for both target molecules at 3 excitation energies: 0.01, 0.39 and 1.00 eV. After maximum of 2 ps of simulation time we observe only four fragmentation channels. Even thought 1HBrT is 0.26 eV less stable the parent ion is observed for all energy spectrum. The fragmentation of 1HBrT is less energy dependent in comparison with 4HBrT. The parent ion of 4HBrT is only presented at 1.00 eV of E_{exc}, while the whole spectrum is dominated by neutral H-loss which decreases as E_{exc} increases. The inverse charge distribution is observed for m/z=68 and m/z=79 for all E_{exc} i.e. Br^- loss is more probably for 1HBrT, while negatively charged triazole is more prominent for 4HBrT (m/z=68 amu).

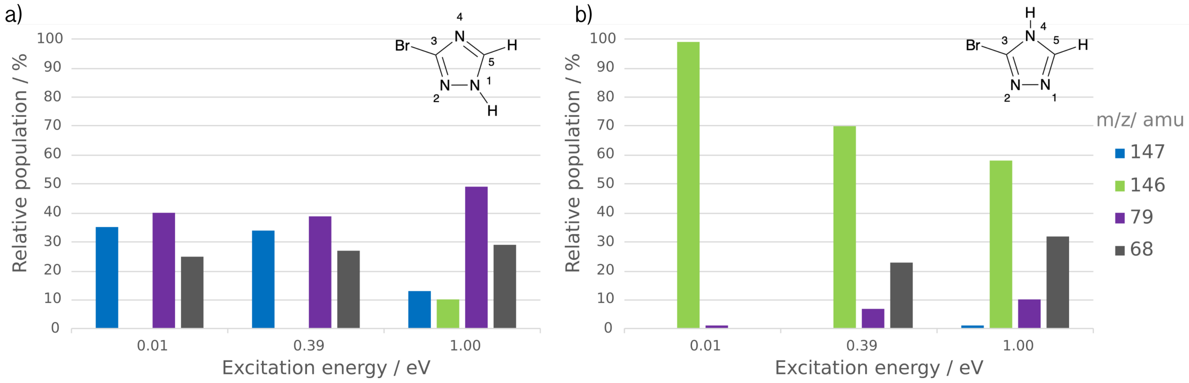

Figure S11: *ab initio* molecular dynamics simulations for 1HBrT and 4HBrT. Relatvie channel population in % is shown as a function of excitation energy for a given fragmentation channel represented as charge-over-mass ratio, m/z in atomic mass unit, amu.

2.3 Benchmark

To assure the qualitative accuracy of the molecular dynamics simulations we benchmark the relative channel populations based on the static calculations i.e. comparing the energy thresholds for given fragmentation channel at different levels of theory: including geometries and energies at i) B3LYP/def2-SVP and ii) B3LYP/aug-cc-PVTZ levels of theory, as well as iii) geometries at B3LYP/def2-SVP and single point (SP) corrected energies at B3LYP/aug-cc-PVTZ i.e. B3LYP/aug-cc-PVTZ//B3LYP/def2-SVP. In most cases the relative energy populations ΔΔE are similar for SP corrected structures and unexpectedly similar to lower level of theory. In addition to the most stable minima for negatively charge fragments and neutrals shown in Fig. S3 we calculated other isomers. Thus, we show various channels of CN, CN_2, HBr and CN^- emissions.

Table S1: Relative intensities of anion fragments formed upon electron attachment to 1HBrT & 4HBrT and the threshold energies calculated at the b3lyp/aug-cc-pVTZ level of theory.

Anion	Relative Intensity / %		Neutral products	Calculated exit channels / eV		ΔΔ(1H-4H) / eV
	1HBrT	4HBrT		1HBrT	4HBrT	
M^-	9.2	10	-	0.04	0.04	0.00
$[M\text{-}H]^-$	0.86	3.22	H	0.77	0.51	0.26
			H	2.25	2.34	-0.09
$[M\text{-}CN]^-$	0.11	0.03	CN	0.19	2.96	-2.77
			CN	5.84	1.68	4.16
			CN	3.71	3.53	0.18
			CN	1.99	-0.08	2.07
$[M\text{-}CN2]^-$	0.13	0.04	CN_2	3.02	2.78	0.24
			CN_2	3.45	3.35	0.09
Br^-	81.5	37.9	[M-Br]	-0.01	-0.04	0.02
$[M\text{-}HBr]^-$	8	48.7	HBr	2.10	2.11	-0.01
			HBr	0.70	0.44	0.26
CN^-	0.2	0.05	[M-CN]	1.36	1.43	-0.07
			[M-CN]	3.81	1.43	2.38
			[M-CN]	1.92	1.21	0.71
			[M-CN]	0.52	0.25	0.26

Table S2: Relative intensities of anion fragments formed upon electron attachment to 1HBrT and 4HBrT, along with the differences in threshold energies calculated at b3lyp/aug-cc-pVTZ, and single-point energy calculations using b3lyp/aug-cc-pVTZ on the top of the structure optimized with b3lyp/def2-SVP i.e. b3lyp/aug-cc-pVTZ//b3lyp/def2-SVP.

Anion	Relative Intensity / %		Neutral products	ΔΔ(1H-4H) / eV		
	1HBrT	4HBrT		b3lyp/aug-cc-pVTZ//b3lyp/def2-SVP	b3lyp/def2-SVP	b3lyp/aug-cc-pVTZ
M^-	9.2	10	-	0.63	0.74	0.00
$[M-H]^-$	0.86	3.22	H	0.26	0.30	0.26
			H	-0.09	-0.11	-0.09
$[M-CN]^-$	0.11	0.03	CN	2.07	2.06	2.07
			CN	-2.88	-2.85	-2.77
			CN	0.17	0.33	0.18
			CN	4.15	4.20	4.16
$[M-CN2]^-$	0.13	0.04	CN_2	0.26	0.08	0.24
			CN_2	0.12	0.05	0.09
Br^-	81.5	37.9	[M-Br]	0.02	0.01	0.02
$[M-HBr]^-$	8	48.7	HBr	0.01	0.01	-0.01
			HBr	0.26	0.30	0.26
CN^-	0.2	0.05	[M-CN]	0.26	0.30	0.26
			[M-CN]	-0.07	-0.01	-0.07
			[M-CN]	0.71	0.74	0.71
			[M-CN]	2.37	2.51	2.38

Table S3: Threshold energies (in eV) for anion fragments formed upon electron attachment to 1HBrT and 4HBrT, optimized at b3lyp/def2-SVP, and single-point energy calculations using b3lyp/aug-cc-pVTZ on the top of the structure optimized with b3lyp/def2-SVP i.e. b3lyp/aug-cc-pVTZ//b3lyp/def2-SVP. Key comparisons include the threshold energies of 1HBrT and 4HBrT using the same basis set.

Anion	b3lyp/def2-SVP		b3lyp/aug-cc-pVTZ//b3lyp/def2-SVP		ΔΔ(1H-4H)	
	1HBrT	4HBrT	1HBrT	4HBrT	b3lyp/def2-SVP	b3lyp/aug-cc-pVTZ//b3lyp/def2-SVP
M^-	0.27	-0.47	-0.34	-0.97	0.74	0.63
$[M-H]^-$	1.23	0.94	0.77	0.51	0.30	0.26
	2.77	2.88	2.25	2.34	-0.11	-0.09
$[M-CN]^-$	1.30	4.14	0.18	3.06	-2.85	-2.88
$[M-CN2]^-$	4.12	4.20	3.02	3.19	0.08	0.26
Br^-	0.97	0.97	-0.01	-0.03	0.01	0.02
$[M-HBr]^-$	2.84	2.83	2.10	2.10	0.01	0.01
	1.39	1.09	0.71	0.44	0.30	0.26
CN^-	1.64	1.34	0.51	0.25	0.30	0.26

2.4 Analytic Continuation Calculation for the Lowest Lying Resonances

The methods of analytic continuation is applied here. An essence of this class of methods is based on introduction of an perturbation potential λV to the molecular Hamiltonian

$$H \to H + \lambda V \; . \tag{1}$$

The one-electron potential employed in this case is represented by the charges positioned on all of the nuclei of the molecule as

$$\lambda V(\mathbf{r}) = \lambda \sum_{i=1}^{8} \frac{1}{|\mathbf{r} - \mathbf{R}_i|} \; , \tag{2}$$

where $\mathbf{R}_i$ are positions of the 3 nitrogen, 2 hydrogen, 2 carbon and 1 bromine atoms. The application (1) of the perturbation potential transfers the continuum resonance states into the bound space where the quantum chemistry techniques can be used. In this case we employed the aug-cc-pVTZ basis set and the CCSD method to treat the electronic correlation to make it compatible with the other calculations presented in the paper. Fig.S12 displays the singly occupied molecular orbital (SOMO) of the main reference of the CCSD method employed, for the two tautomers considered here.

Table S4: Lowest resonance positions and widths for 1HBrT and 4HBrT. There are 2 methods of analytical continuation applied: the older RAC method,[10] and the more accurate recent barycentric approach BAC.[11]

	1HBrT		4HBrT	
Method	Energy E_r (meV)	Width Γ (meV)	Energy E_r (meV)	Width Γ (meV)
RAC[10]	244	18	197	2
BAC[11]	251	5	201	0.1

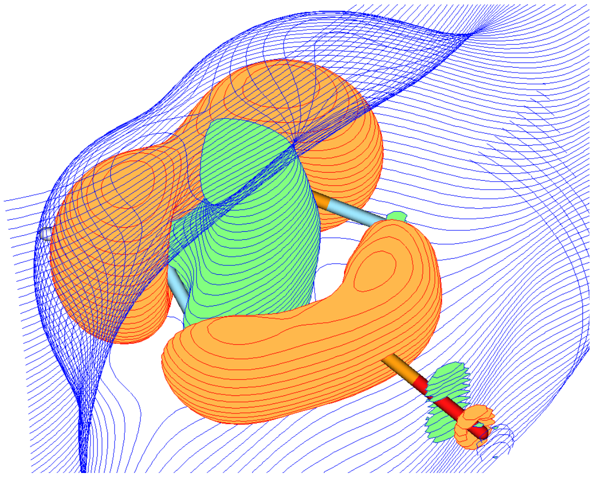

(a) 1HBrT

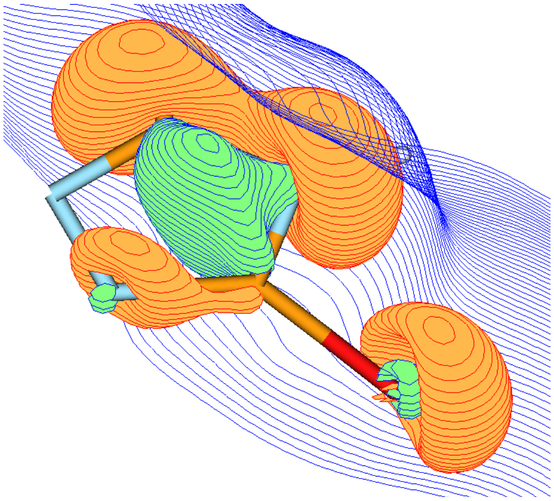

(b) 4HBrT

Figure S12: Resonant state's SOMO of the main reference used by the present CCSD method. Left panel a) is for the 1HBrT, while the right panel b) is for the 4HBrT.

References

(1) Saqib, M.; Izadi, F.; Isierhienrhien, L. U.;Ončák, M.; Denifl, S. Decomposition of Triazole and 3-Nitrotriazole Upon Low-Energy Electron Attachment. *Phys. Chem. Chem. Phys.* **2023**, *25*, 13892–13901.

(2) TURBOMOLE V7.3 2018, a development of University of Karlsruhe and Forschungszentrum Karlsruhe GmbH, 1989-2007, TURBOMOLE GmbH, since 2007; available from
`http://www.turbomole.com`.

(3) Weigend, F. A Fully Direct RI-HF Algorithm: Implementation, Optimised Auxiliary Basis Sets, Demonstration of Accuracy and Efficiency. *Physical Chemistry Chemical Physics* **2002**, *4*, 4285–4291.

(4) Grimme, S. Accurate Description of Van der Waals Complexes by Density Functional Theory Including Empirical Corrections. *Journal of computational chemistry* **2004**, *25*, 1463–1473.

(5) Grimme, S.; Ehrlich, S.; Goerigk, L. Effect of the Damping Function in Dispersion Corrected Density Functional Theory. *Journal of computational chemistry* **2011**, *32*, 1456–1465.

(6) Becke, A. D. A New Mixing of Hartree–Fock and Local Density-Functional Theories. *The Journal of Chemical Physics* **1993**, *98*, 1372.

(7) Lee, C.; Yang, W.; Parr, R. G. Development of the Colle-Salvetti Correlation-Energy Formula Into a Functional of the Electron Density. *Phys. Rev. B* **1988**, *37*, 785–789.

(8) Becke, A. D. Density-Functional Thermochemistry. IV. A New Dynamical Correlation Functional and Implications for Exact-Exchange Mixing. *The Journal of Chemical Physics* **1996**, *104*, 1040.

(9) Weigend, F.; Ahlrichs, R. Balanced Basis Sets of Split Valence, Triple Zeta Valence and Quadruple Zeta Valence Quality for H to Rn: Design and Assessment of Accuracy. *Physical Chemistry Chemical Physics* **2005**, *7*, 3297–3305.

(10) Horáček, J.; Paidarová, I.; Čurík, R. On a simple way to calculate electronic resonances for polyatomic molecules. *The Journal of Chemical Physics* **2015**, *143*, 184102.

(11) Čurík, R.; Horáček, J. Determination of electronic resonances by analytic continuation using barycentric formula. *Computer Physics Communications* **2025**, *306*, 109379.
